# Retraction

**DOI:** 10.1590/S1984-29612024048

**Published:** 2024-08-19

The editorial board of Brazilian Journal of Veterinary Parasitology communicates the formal publication of Retraction for extracting the article:

Pinheiro RHS, Bezerra AM, Giese EG. Morphological identification of *Skrjabinisakis* Mozgovoi, 1951 (Nematoda: Anisakidae) in *Kogia sima* (Cetacea: Kogiidae) from Brazilian waters. *Rev Bras Parasitol Vet* 2023; 32(4): e013423. https://doi.org/10.1590/S1984-29612023064.

The article is being retracted because the aforementioned article, apparently, it was produced with biological samples, in theory, used without authorization from the teams responsible for dealing with the stranding of the cetacean.

Profa. Dra. Rosangela Zacarias Machado

Editor-chief

